# The Role of Galectins in Cervical Cancer Biology and Progression

**DOI:** 10.1155/2018/2175927

**Published:** 2018-05-08

**Authors:** Lufang Wang, Yanyan Zhao, Yanshi Wang, Xin Wu

**Affiliations:** Department of Gynecology, The First Affiliated Hospital of China Medical University, Shenyang, Liaoning, China

## Abstract

Cervical cancer is one of the malignant tumors with high incidence and high mortality among women in developing countries. The main factors affecting the prognosis of cervical cancer are the late recurrence and metastasis and the effective adjuvant treatment, which is radiation and chemotherapy or combination therapy. Galectins, a family containing many carbohydrate binding proteins, are closely involved in the occurrence and development of tumor. They are involved in tumor cells transformation, angiogenesis, metastasis, immune escape, and sensitivity against radiation and chemotherapy. Therefore, galectins are deemed as the targets of multifunctional cancer treatment. In this review, we mainly focus on the role of galectins, especially galectin-1, galectin-3, galectin-7, and galectin-9 in cervical cancer, and provide theoretical basis for potential targeted treatment of cervical cancer.

## 1. Introduction

As the second most common female malignant tumor around the world, cervical cancer occupies the second place of fatality in gynecological oncology in developing countries [1]. The present studies showed that cervical cancer is closely associated with the infection of high risk human papillomavirus (HPV), but there are still a large number of patients with cervical cancer who are not infected with HPV, suggesting that other factors such as cell genetic changes can also lead to disease progression [2]. The main treatments for cervical cancer are surgery (including pelvic lymphadenectomy and radical hysterectomy), radiotherapy, and chemotherapy. Both radical hysterectomy and radiotherapy are considered curative for localized disease, while for advanced cancer, concurrent radiochemotherapy remains a cornerstone intervention [3]. Targeted therapy is becoming a hot spot of research in recent years and the current clinical targeted therapies primarily for the treatment of cervical carcinoma are EGFR [4, 5] and COX-2 [6, 7]. However, the survival rate of cervical cancer did not increase significantly but increased with adverse events. Therefore, further prognosis biomarkers and therapeutic targets research need to be carried out.

**Figure 1:**
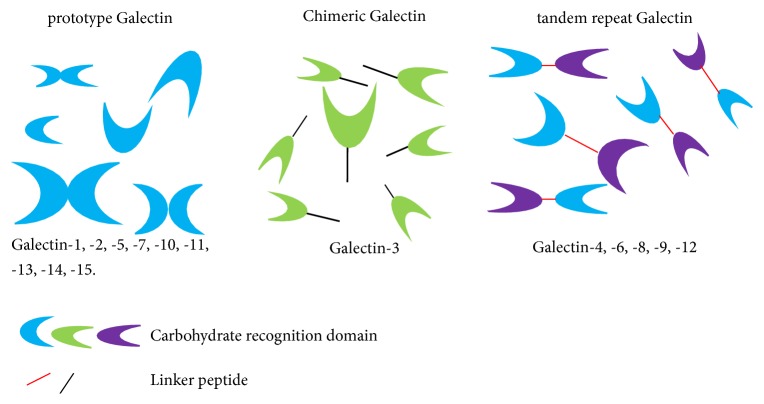
Galectins are divided into three types according to their molecular structure: (1) prototype galectin (galectin-1, galectin-2, galectin-5, galectin-7, galectin-10, galectin-11, galectin-13, galectin-14, and galectin-15); (2) chimeric galectin (galectin-3); and (3) tandem repeat galectin (galectin-4, galectin-6, galectin-8, galectin-9, and galectin-12).

Galectins, a family of *β*-galactoside binding proteins, widely exist not only in animals but also in bacteria and fungi at different levels. They are a highly conserved core sequence containing 130 amino acids and a carbohydrate recognition domain (CRD). There are two typical characteristics of galectins protein family: (1) sharing significant similarities in a conserved amino acid sequence; (2) a high affinity for beta galactoside sugars [8]. It has been discovered and named that galectins (Gals) in mammals have 15 subtypes. According to their molecular structure, they are divided into three types: (1) prototype galectins: prototype galectins are proteins containing a single carbohydrate recognition domain (CRD), which often forms homodimers and includes galectin-1, galectin-2, galectin-5, galectin-7, galectin-10, galectin-11, galectin-13, galectin-14, galectin -15; (2) chimeric galectins: chimeric galectins contain two domains, one C-terminal CRD and one noncarbohydrate-binding N-terminal domains self-associating into oligomers, and only include galectin-3; (3) tandem-repeat galectins: tandem-repeat galectins are dimers consisting of two CRDs connected by a linker peptide and include galectin-4, galectin-6, galectin-8, galectin-9, galectin-12 [9] (Figure 1). Galectins are reported to have multiple roles in different parts of the cells, such as cell membrane, cytoplasm, extracellular matrix, and the intracellular receptors which are numberous, such as cytokeratins, cyclins, transcription factors, Bcl-2, and h-rask-ras [10, 11]. Galectins can be secreted into extracellular matrix [12]. Since galectins lack signal peptide, they can only be synthesized in the cytoplasm and then secreted to the outside of the cell by the vesicles directly, the endoplasmic reticulum and Golgi apparatus [13]. The special way of secretion can prevent galectins from adhering to new generation of glycoprotein oligosaccharide prematurely [11]. Some factors affect the secretion of galectins, such as components of the extracellular matrix and inflammatory factors [14]. Galectins on the cell surface, the secretion of extracellular matrix, and cellular and extracellular are closely related to cell adhesion and signal transduction [15]. Galectins outside the cells combine to diverse cell surface receptors forming as carbohydrates [16, 17]. Therefore, not only in the aspects of tumorigenesis and development, but also in terms of organogenesis and connective tissue diseases, galectins all play an important role [18].

Many previous experimental researches showed that galectins play an essential role in the origin and development of cancer, such as angiogenesis [19], cell adhesion, invasion, and migration [20]. However, the role and mechanism of the same galectin is different in different tumors. For example, upregulated Gal-3 contributed to increased cancer cell migration and motility through downregulating K-Ras-Raf-Erk1/2 pathway in colon cancer [21, 22]. In gastric cancer, Gal-3 may promote metastasis by enhancing the expression of MMP-1 and protease-activated receptor-1 (PAR-1) [23]. In vitro as well as in vivo studies had indicated tumor suppressive effect of galectin-7 on colon cancer [24]. Even in the same kind of tumor, the role of different galectins is not also the same; for example, galectin-1 is overexpressed and can increase metastasis of colorectal cancer [25] while Gal-4 is downregulated in colorectal cancer [26]. Gordower et al. found that the level of Gal-3 expression significantly decreases in astrocytic tumors from low grade to high grade while some highly malignant tumor cells increase expressed higher than normal tissue [27]. Studies showed that the expression of galectins changes a lot in cervical cancer [28, 29]. But there is no review on the relationship between cervical cancer and galectins. In the review, we focus on the biological role of galectins in the development of cervical cancer and their potential role in targeted therapy of cervical cancer.

## 2. Galectins in Cancer

The activation of protooncogenes to the occurrence and development of cancer entails a lot of complex processes that involves multiple factors. These factors are dependent on genetic changes, external cellular pressures, function and regulation of the body's immune system, and the microenvironment of the tissue [30]. Studies showed that most galectins are involved in the development of various cancers [31, 32] and the processes they involved in are mainly tumor cell transformation through interacting with oncogenes such as HRAS and KRAS [33, 34]. It has been found that galectins not only participate in cell cycle and cell apoptosis [35] but also are involved in the development of tumor through tumor immune escape, tumor metastasis, and tumor angiogenesis [36].

### 2.1. The Role of Galectins in Cancer Cell Proliferation

The proliferation and metastasis of malignant tumor cells are the important factors that influence the treatment of tumor. Therefore, understanding the factors and mechanism that influence and regulate malignant tumor cells proliferation and metastasis is of great significance for early diagnosis and clinical treatment of tumors.

In tumor cell experiments, overexpression of Gal-1 will stimulate cell proliferation in human glioma cells [37] and thyroid cancer [38]. Mercier et al. [39] showed that Gal-1 mainly regulates cell cycle through Ras/Raf/ERK2 signaling pathway. Gal-3 also plays a tumor promotion role in the proliferation of cancers cells. Gal-3 can increase cell growth of hepatocellular carcinoma [40], glioma cells [41], pancreatic cancer proliferation, The effects of Gal-3 on the cell proliferation and cycle came about as a result of the translocation ability of Gal-3 into the nucleus by binding with Impotin, Sufu, and Nup98, wherein it controls the cell cycle through the interaction with cyclin A, cyclin D, cyclin E, p21 (WAF1), and p27 (KIP1), accelerating cancer cell proliferation [42]. Studies performed on diverse cell types, mostly cancerous cell lines, have demonstrated that galectin-7 has a suppressive effect on cell proliferation. Indeed, ectopic expression or addition of exogenous galectin-7 in the DLD-1 human colon carcinoma cell line [24] and the neuroblastoma cells SK-N-MC, respectively [43], drastically reduced tumor cell proliferation. Studies performed on diverse cell types, mostly cancerous cell lines, have demonstrated that galectin-7 has a suppressive effect on cell proliferation. The molecular mechanism by which galectin-7 participates in cell proliferation remains to be clarified. However, galectin-7 could be an effector of the tumor suppressor gene p53. Strikingly, galectin-7 expression is strongly induced by p53 [44] and lack of wild type p53 in human keratinocytes cell lines prevents galectin-7 expression induction in response to UVB irradiation [45]. Gal-9 can induce cell proliferation in human osteoblasts through sc-Src-ERK and NFkB signals pathway [46] (Table 1).

### 2.2. The Role of Galectins in Cancer Cell Apoptosis

Cancer is a disease with abnormal cell proliferation and cell death. Solid tumors cells mainly have two death forms, necrosis or apoptosis. Cell apoptosis not only plays a significant role in tumorigenesis and development but comes to play when chemotherapy, radiotherapy, and biological therapy are used to treat cancers [47]. One way to treat cancer is to control or possibly stop the uncontrolled growth of cancer cells. One way to treat cancer is to control or possibly stop the uncontrolled growth of cancer cells. Since apoptosis avoidance is a characteristic of cancer, targeted apoptosis may become the most successful nonsurgical treatment. Nowadays, many antitumor drugs are aimed at different endogenous and exogenous pathways [48–50]. Two commonly used therapeutic targeting strategies are stimulating apoptotic molecules and inhibiting antiapoptotic molecules [51]. Some of the targets that have been researched include ligands for death-receptors, XIAP inhibition [52], inhibitors for BCL-2 [53], and alkylphospholipid analogs (APL) which act as apoptotic signals [49]. Studies showed that Gal-1 binding induces apoptosis through triggering apoptotic pathways (induction of the activation protein-1 (AP-1) transcription factor and then the activation of apoptotic pathway promoters caspase-8 and caspase-3 and subsequently downregulation of mitochondrial Bcl-2) [54] and promoting TCR chain phosphorylation [55]. On the contrary, Gal-3 can suppress cells apoptosis and the antiapoptotic activity is given by a functional antideath (NWGR) motif, an conserved amino acid sequence located in the BH1 domain of the Bcl2 gene family [56]. Gal-3 combines with CD95/Fas and subsequently suppresses the activation of CD95/Fas-mediated caspase-8 [57]. The inhibitory effect of Gal-3 on cell apoptosis is regulated by the phosphorylation/dephosphorylation of ser-6 residues, which acts as a “switch” in binding Gal-3 to ligands [58]. Gal-7 and Gal-9 tend to display proapoptotic effects. Moisan et al. [59] have shown that Gal-7 plays a promoter role in tumor cells apoptosis through activating mitochondrial cytochrome c release and JNK activity. Gal-9 also induces apoptosis through activating JNK and p38-MAPK pathway and subsequently activating caspase-3, caspase-8, and caspase-9 in multiple myeloma cells [60]. (Table 1).

### 2.3. Metastasis: Invasion and Adhesion

Tumor invasion and metastasis are the unique characteristics of malignant tumor compared with benign tumor and they are also the most important factors that affect treatment and prognosis. There are many essential steps that carry out cancer cells metastasis. Firstly, the connection between the cells became loose; cancer cells invade to extracellular matrix through interactions among cells and adhesion to vascular endothelium. Then the invaded cancer cells moved to distant sites through hemokinesis and proliferate by forming new blood vessels and then transfer to other place [61]. The cytokines involve tumor endothelial cell interactions that occur between various surface adhesion molecules including integrins, ICAM-1, VCAM-1, or selectins [62]. Studies showed that Gal-1 binding with CD44 and CD326 promotes tumor cells metastasis to cell matrix and adhesion to vascular endothelial cells and knockdown of Gal-1 significantly reduced their lung metastatic potential in colon and breast cancer [63, 64]. Merseburger et al. found that Gal-3 was highly expressed in clear cell renal carcinoma (CC-RCC) especially in those patients with distant metastasis [65]. O'Driscoll et al. confirmed that overexpression of Gal-3 can increase the adhesion between lung cancer cells to extracellular matrix, leading to cell motility and invasiveness, and inhibiting Gal-3 by lactose will significantly weaken this effect [66]. Matarrese et al. found that Gal-3 promotes invasiveness of human breast carcinoma cells through interaction with the *α*6*β*1 integrin [67]. Iurisci et al. [68] also showed that higher Gal-3 in blood of cancer patients could induce the vascular endothelial cells to secrete more cytokines like colony-stimulating factor (G-CSF) and interleukin-6 (IL-6), leading to interactions among the cytokines, vascular endothelial and cancer cells, and ultimately increasing endothelial cells migration and tubule formation. Demers et al. [69] showed that through regulating metastatic genes like metalloproteinase 9 (MMP9) Gal-7 suppresses the invasion of lymphoma cells. Gal-9 can inhibit invasion and metastasis of tumor cells and is a protective factor affecting prognosis. Transfected with Gal-9 in colon cancer and melanoma models can significantly decrease the risk of metastasis [70]. Kageshita et al. [71] also confirmed that downregulation of gal-9 in hepatocellular carcinoma cells (HCC) could significantly suppress the risk of lymph node metastasis, vascular invasion, and intrahepatic metastasis, improving survival rate of patients. They also analyzed how Gal-9 inhibited invasion and metastasis of HCC and showed that Gal-9 blocked cancer cells adhesion to extracellular matrix (ECM) through downregulation of ECM components such as collagen, laminin, and fibronectin (Table 1).

### 2.4. The Role of Galectins in Tumor Angiogenesis

Angiogenesis plays an important role for the development and evolution of tumor, since the rapid expansion of tumor requires continuous new blood vessels, which is very important for the growth and metastasis of tumor cells. Ito et al. [72] reported that adding exogenous Gal-1 increases the capillary-like tube forming capacity of endothelial cells within basal membrane Matrigel cultures. The extracellular Gal-1 structurally promotes tumor angiogenesis by reinforcing and stabilizing connections of vascular endothelial cells and extracellular matrix interactions within the tumor microenvironments. Studies showed that Gal-1 can induce angiogenesis and thiodigalactoside can block effects of Gal-1 through suppressing angiogenesis and protection against oxidative stress. As a chemoattractant inducting epithelial cells transfer to vascular endothelial cells in vitro and in vivo, Gal-3 is critical for tumor angiogenesis [73]. Markowska et al. [74] found that Gal-3 is involved in VEGF- and bFGF-mediated angiogenesis and through binding with GnTV-modified N-glycans on *α*v*β*3 integrin, they activate FAK-mediated signaling pathways which modulate endothelial cell migration in the angiogenic cascade. Their further study [75] showed that Gal-3 also binds to VEGFR2 to increase angiogenic response to VEGF-A. Another study showed that the combined action of Gal-1 and Gal-3 can increase effect on angiogenesis via the activation of VEGFR1 and the decrease receptor endocytosis [76]. Gal-9 appears to be the only galectin with an inhibitory effect on angiogenesis. It remains to be established which mechanisms underlie this inhibitory effect [77]. Thus far, the angiostimulatory activity of galectins has been linked to several signaling pathways, including the VEGF/VEGFR2 signaling axis, integrin signaling, and Ras signaling [78, 79] The ability of galectin-1, galectin-3, and galectin-8 to trigger these signaling pathways has been linked to cross-linking of different receptors like VEGFR2, neuropilin-1, beta-integrins, and CD166 [80]. Whether galectin-9 also cross-links these receptors on endothelial cells remains to be established. In fact, most of the receptors found to interact with galectin-9 appear to be predominantly involved in immune cell activity and function, for example, T-cell immunoglobulin mucin 3 (TIM-3), cell surface bound protein disulfide isomerases (PDI), CD40, and CD44 [81]. Interestingly, since several of these receptors are also expressed by endothelial cells, it could be hypothesized that some are involved in regulating the effects of galectin-9 in angiogenesis (Table 1).

### 2.5. The Role of Galectins in Immune Responses

In the traditional sense, the immune cells play an important role of monitoring the occurrence of tumor through a series of mechanisms from the host immune attack. T lymphocytes, natural killer (NK) cells and cytokines induced Killer cells (CIK), dendritic cells (DC) and DC-CIK cells, and so on all have certain antitumor activity but the body's core of tumor immunotherapy worker is T lymphocytes. The role of galectins in immune responses is mainly through regulating levels of activated effector NK cells and T cells. Gal-1 selectively deletes TH1 and TH17 cells and promotes the proliferation of CD4+CD25+Foxp3+ regulatory T cells (Tregs), further enhancing immunosuppressive activity. Gal-3 can keep the distance between T-cell receptor (TCR) and CD8 molecule, causing the latter inactivation and through inhibiting the interaction between the heavily* O*-glycosylated tumor-derived complex class I-related chain A (MICA) and NK cells, damaging the function of the latter cells, eventually leading to immune evasion. Gal-1 promotes cancer progression through inhibiting immune responses. Perillo et al. [82] found that Gal-1 induced the apoptosis of activated T cells and their further study [83] revealed that cell surface glycoproteins on activated T cells were the receptors for extracellular Gal-1, such as CD2, T-cell receptor (TCR), and CD95. On the other hand, Gal-1 exposure significantly promotes the differentiation of Treg cells (CD4+CD25+FoxP3+) [84]. Gal-1 treatment in vivo greatly increases IL-10 production inducing T cells that suppress autoimmune inflammation [85]. Gal-3 was also regarded as one of targeting molecules involved in the immune escape in the progression of cancers [86]. Stillman et al. [87] found that exogenous Gal-3 causes apoptosis in activated T cells. Further study [88] also revealed that Gal-3 suppressed the binding of MHC class I chain-related molecular, resulting in the impairment of the NK cell activation. Labrie et al. found that Gal-7 expression was induced by mutant p53. Gal-7 not only increased the invasive behavior of ovarian cancer cells by inducing MMP-9 and increasing cell motility but also has immunosuppressive properties by killing Jurkat T cells and human peripheral T cells [89]. Gonçalves et al. [90] found that a fundamental molecular pathway, which includes ligand-dependent activation of ectopically expressed latrophilin 1 and possibly other G-protein coupled receptors leading to increased translation and exocytosis of the immune receptor Tim-3 and its ligand galectin-9. This occurs in a protein kinase C and mTOR- (mammalian target of rapamycin-) dependent manner. Tim-3 participates in galectin-9 secretion and is also released in a free soluble form. Galectin-9 impairs the anticancer activity of cytotoxic lymphoid cells including natural killer (NK) cells. Soluble Tim-3 prevents secretion of interleukin-2 (IL-2) required for the activation of cytotoxic lymphoid cells. These results were validated in ex vivo experiments using primary samples from acute myeloid leukemia (AML) patients. This pathway provides reliable targets for both highly specific diagnosis and immune therapy of AML (Table 1).

### 2.6. The Role of Galactins in the Treatment of Cancer

At present, the therapy of cancer mainly concentrated on surgery, radiotherapy, chemotherapy, immunotherapy, and molecular targeted therapy. Studies [91] have shown that hypoxia can significantly reduce the effectiveness of radiation, chemotherapy, and molecular targeted therapy and verified that Gal-1 derived by tumor binding with N-glycans is important in hypoxia and tumor angiogenesis and they also found that hypoxia can induce secretion of Gal-1 by Kaposi's sarcoma cells via NFkB signal pathway. Zhao et al. [92] also showed that the expression of Gal-1 was also upregulated by the level of HIF-1*α* stabilization within tumors and hypoxia-responsive elements are located at −441 to −423 bp upstream of the transcriptional start site of the Lgals1 gene and are essential for HIF-1-mediated galectin-1 expression. Other studies [93] also revealed that hypoxia-exposed cancer cells produce the higher levels of Gal-1, which was closely related to HIF-1*α* and carbonic anhydrase IX (CA IX) and knocking down Gal-1 can reduce hypoxia-induced invasion and migration of cancer cells. The studies also showed that the increased Gal-1 by hypoxia is related to poor prognosis of cancer patients. The similar study [94] found that Gal-3 was also upregulated within hypoxic regions of murine and human melanomas. Currently, molecular targeted therapy mainly focused on tumor angiogenesis or intratumoral hypoxia-induced protumorigenic signaling. Many research [95, 96] have demonstrated that using antiangiogenic drugs could improve the outcomes of chemotherapy or immunotherapy. The studies [97, 98] investigated that the treatments combining Gal-1 inhibition with other cancer therapies showed that anginex (a novel angiogenesis inhibitor) could improve radiochemotherapy outcomes in transgenic model of aggressive breast cancer and A squamous cell (SCCVII) xenograft tumor mouse model, but the detailed mechanism involved was still in research. Croci et al. [79] showed that vessels in anti-VEGF-sensitive tumors express more *α*2-6-linked sialic acid reducing the secretion of Gal-1. In contrast, anti-VEGF refractory tumors secreted more galectin-1. Garín et al. [84] found that using MMP inhibitors targeting on cleavage of Gal-3 significantly reduced angiogenesis in breast cancer. Grosset et al. [99] found that cytosolic galectin-7 impaired p53 functions and induced chemoresistance in breast cancer cells by affecting mitochondrial transport. Matsukawa et al. [100] used 68 clinical tissues from 18 patients with oral squamous cell carcinoma (OSCC) who received radiotherapy and chemotherapy followed by surgery and detected the expression of galectin-7 by proteomic analysis and immunohistochemical analysis. They found that the sensitivity and specificity of the galectin-7 prediction score (G7PS) in predicting this resistance were of 96.0% and 39.5%, respectively, in the 68 test cases. The cumulative 5-year disease-specific survival rate was 75.2% in patients with resistant prediction using G7PS and 100% in patients with sensitive prediction. They found that low galectin-7 expression is more likely to exhibit chemotherapy and/or radiotherapy resistance, suggesting that galectin-7 is a potential predictive marker of chemotherapy and/or radiotherapy resistance in patients with OSCC. However, the exact mechanism is still unclear (Table 1).

## 3. Galectins in Cervical Cancer

The current research about the relationship between cervical cancer and galectins mainly concentrated on tumor formation, angiogenesis, radiation, chemotherapy sensitivity, and so on [101, 102]. Gal-1, Gal-3, Gal-7, and Gal-9 have been reported in cervical cancer.

### 3.1. Galectin-1 in Cervical Cancer

Gal-1 has been shown to be involved in different steps of cancer cell invasion and metastasis by regulating cell adhesion and cell migration [103]. In ovarian and prostate cancer cell lines, Gal-1 promotes the adhesion of tumor cells to the extracellular matrix [104]. Kohrenhagen et al. [105] examined the expression of galectin-1 that was examined in 80 formalin-fixed, paraffin-embedded cervical tissues: 20 benign cervical specimens, 20 low-grade squamous intraepithelial lesions (LGSIL), 20 high-grade squamous intraepithelial lesions (HGSIL), and 20 invasive squamous cell carcinomas (ISCC). They found that the intensity of the galectin-1 expression on stromal cells next to the transformed cells increased according to the pathologic grade: benign cervical tissue, LGSIL, HGSIL, and ISCC. The epithelial cells were always negative for galectin-1. These results suggest that galectin-1 expression on stromal cells increases with the histopathologic grade of cervical tissues. In the study of Kim et al. [106], immunohistochemical analysis revealed that galectin 1 expression was found in most peritumoral stroma samples (72/73; 98.6%). Galectin 1 expression was significantly correlated with the depth of invasion in the cervix and lymph node metastasis on univariate analysis. When progression-free survival of all of the patients studied was analyzed based upon galectin 1 expression, galectin 1 expression was not correlated with progression-free survival. Punt et al. [28] analyzed immunohistochemistry combined with clinical data of 155 patients (including 41 relapses and 30 deaths) and found that, as an independent predictor, strong Gal-1 expression was closely related to invasion and metastasis in cervical cancer and an independent predictor for poor survival and a likelihood of receiving postoperative radiotherapy. The functional effects of gal-1 on cervical cancer cells are mainly characterized by promoting cell proliferation and invasion. Kim et al. [106] downregulation of galectin 1 using small interfering RNA resulted in the inhibition of cell growth and proliferation of HeLa and SiHa cells. Moreover, the ability of cells to invade was significantly reduced by galectin-1 small interfering RNA. They chose HeLa and SiHa cells to do the galectin 1 small interfering RNA because in both cell lines galectin 1 was overexpressed. Gal-1 also has an effect on the radiotherapy and chemotherapy of cervical cancer. Huang et al. [107] revealed that Gal-1 is an independent prognostic factor associated with local recurrence in cervical cancer patients undergoing definitive radiation therapy. Gal-1 also plays an important role in the radiation therapy. The same group in further studies [108] found that Gal-1 mediates radioresistance mainly through two factors: The first factor is DNA repair. Parliament and Murray [109] reviewed the clinical evidence of numerous DNA repair genes involving radioresistance. H-Ras involves DNA repair and radioresistance [110]. In addition, galectin-1 can interact with H-Ras to promote downstream signals [111]. The second factor is tumor hypoxia. Hypoxia is a radioresistance-influencing factor for patients with cervical cancer. Coincidentally, hypoxia is associated with galectin-1 expression [92]. These findings suggest that Gal-1 mediates radioresistance through the H-Ras-dependent pathway involved in DNA damage repair and targeting Gal-1 may improve the local control of cervical cancer (Table 2).

### 3.2. Galectin-3 in Cervical Cancer

Galectin-3 plays a role in a variety of physiological and pathological processes. Studies of pancreatic [112], gastric carcinomas [113] found galectin-3 to be upregulated in these tissues compared to normal tissues. Studies conducted by Schoeppner et al. [114] also revealed that galectin-3 expression is related to neoplastic transformation and progression towards metastasis in colon carcinoma. Lee et al. [115] showed that Gal-3 was downregulated in cervical cancer tissues compared to normal tissues and gradually decreased in accordance with the histopathologic grades from LSIL (low-grade squamous intraepithelial lesions) to HSIL (high-grade squamous intraepithelial lesions) and to ISCC (invasive squamous cell carcinomas). Punt et al. [28] found that weak and positive tumor cell galectin-3 expression was correlated with increased and decreased tumor invasion. Study conducted by Balasubramanian et al. [116] through using antigalectin-3 based ELISA and agglutination assays detected galectin-3 level of stages I–V of cervical cancer and found that stage dependent expression of galectin-3 approx. ranging from 1.0 to 3.3, 4.4 to 5.4, 5.4 to 24.7, 13.1 to 31.9, and 13.9 to 32.9 ng/mg C (creatinine) for stages I–V, respectively, indicating that galectin-3 is closely related to the stage of cervical cancer and galectin-3 may be used as a potential diagnostic tool for monitoring or follow-up of the stage of cancer disease. The research about the effect of Gal-3 on the function of cervical cancer cells is little. Study of Liu et al. [117] using cervical carcinoma cell line SiHa cells, through silencing of Gal-3 expression with specific siRNA largely impaired VEGF-C-enhanced cell invasion, indicating that VEGF-C enhanced cervical cancer invasiveness via upregulation of Gal-3 protein through NF-*κ*B pathway, which may shed light on potential therapeutic strategies for cervical cancer therapy (Table 2).

### 3.3. Galectin-7 in Cervical Cancer

As an endogenous lectin, a fraction of Gal-7 is constitutively localized at the mitochondria. It has been found to interact with the antiapoptotic protein Bcl-2, suggesting its regulatory role in apoptotic processes [118]. Importantly, increased Gal-7 expression has been shown as a positive predictive biomarker for clinical responses after adjuvant radiation therapy in cervical cancer patients [29]. Zhu et al. [119] detected the expression of Gal-7 in normal cervical tissue, CIN I, CIN II, CIN III, and cervical cancer tissues by immunohistochemical method and found that the positive expression rate of Gal-7 in normal cervical tissue, CIN I, CIN II, CIN III, and cervical squamous cell carcinomas were 56.7%, 41.9%, 32.0%, 27.3%, and 25.0%, respectively. The same group also found that the expression of Gal-7 was closely related to the international union of gynecology and obstetrics (FIGO) staging, lymph node metastasis, and 5-year survival rate of cervical squamous carcinoma patients. Higareda-Almaraz et al. [120] found that Gal-7 is downregulated in squamous cervical cancer, high-grade squamous intraepithelial lesions, and cervical cancer cell lines. The results of both researches indicated that Gal-7 was low expression in cervical cancer tissues. The research about the effect of Gal-7 on the function of cervical cancer cells is little. Park et al. [102] showed that, in HeLa cells, human cervical epithelial adenocarcinoma cells, Gal-7 could induce matrix metalloproteinase-9 (MMP-9) through p38 MAPK signaling leading to increasing cell invasion and was associated with immunity ability. Gal-7 also has an effect on the radiotherapy and chemotherapy of cervical cancer. Tsai et al. [29] detected different sensitive proteins to radiation and chemotherapy and found that Gal-7 expressed high in the high sensitive group and Gal-7 expressed low in low sensitive group, which demonstrated that elevated Gal-7 expression is associated with improved outcomes after radiation therapy for cervical cancer (Table 2).

### 3.4. Galectin-9 in Cervical Cancer

Gal-9 exhibits lactose-binding activity [121] and is believed to be involved in cell-cell or cell-matrix interactions. Gal-9 exhibits a variety of biological functions, such as cell aggregation, adhesion, proliferation, and cell apoptosis and modulation of inflammation [122]. However, the expression of Gal-9 has not been fully verified in human tissues without malignancy as well as malignant tumors. Liang et al. [101] detected the expression of Gal-9 and E-cadherin in normal epithelium and endocervical glands, CIN, and cervical cancer tissues by immunohistochemical method and found that Gal-9 and E-cadherin were evidently detected in normal epithelium and endocervical glands, but those in CIN and SCC were significantly faint. Moreover, both the Gal-9 and E-cadherin expressions in HSIL were significantly lower than those in LSIL, suggesting their association with malignant transformation. Punt et al. [28] found that both galectin-3 and galectin-9 expression were significantly correlated with the presence of HPV type 16 or 18 and tumor expression of galectin-9 showed a trend towards improved survival (Table 2).

Galectins play an important role in tumorigenicity, the survival of tumor cells, new blood vessels formation, and tumor metastasis. Moreover, it is involved in adjustment of the immune and inflammatory reaction guidance of the tumor escape immune surveillance. Galectin-1, galectin-3, galectin-7, and galectin-9 may become a prediction to the progression of cervical lesion, valuable markers of prognosis, and basis for target gene therapy in the future.

## Figures and Tables

**Table 1:** Effect of galectins on tumor biology and treatment.

Process	Galectin-1	Galectin-3	Galectin-7	Galectin-9
Proliferation	↑	↑	↓	↑
Apoptosis	↑	↓	↑	↑
Metastasis: invasion and adhesion	↑	↑	↓	↓
Tumor angiogenesis	↑	↑	UNK	↓
Immune responses	↓	↓	↓	↑
Radiotherapy and/or chemotherapy resistance	↑	↑	↓	UNK

UNK, unknown; ↑: increase; ↓: decrease.

**Table 2:** Effect of galectin-1, galectin-3, galectin-7, and galectin-9 on cervical cancer tissue, cell function, HPV infection, and treatment.

Process	Galectin-1	Galectin-3	Galectin-7	Galectin-9
Expression in cervical cancer tissues	↑	↑	↓	↓
Proliferation	↑	↑	↓	UNK
Invasion	↑	↑	↑	UNK
HPV infection	UNK	UNK	UNK	↓
Radiotherapy and/or chemotherapy resistance	↑	↑	↓	UNK

UNK, unknown; ↑: increase; ↓: decrease.
